# Changes in the humoral immunity response in SARS-CoV-2 convalescent patients over 8 months

**DOI:** 10.1038/s41423-020-00605-4

**Published:** 2021-01-08

**Authors:** Pai Peng, Jie Hu, Hai-jun Deng, Bei-zhong Liu, Liang Fang, Kai Wang, Ni Tang, Ai-long Huang

**Affiliations:** 1 Key Laboratory of Molecular Biology for Infectious Diseases (Ministry of Education), Institute for Viral Hepatitis, Department of Infectious Diseases, The Second Affiliated Hospital, Chongqing Medical University, Chongqing, China; 2 Yong-Chuan Hospital, Chongqing Medical University, Chongqing, 402160 China

**Keywords:** Viral infection, Infection

Many countries around the world have seen a sharp rise in COVID-19 cases since the beginning of October due to the second wave of the pandemic. A decline in the antibody response to severe acute respiratory syndrome coronavirus 2 (SARS-CoV-2), which was reported exclusively in the early month, increases the risk of reinfection for convalescent individuals. There is a current need to follow the maintenance of specific antibodies against SARS-CoV-2.

Twenty patients who had recovered from COVID-19 were included in our cohort. Blood samples were obtained in February and October, corresponding to a median of 25 (range 5–33 days) and 230 (range 221–248 days) days after symptom onset (Fig. 1a). Enzyme-linked immunosorbent assay was performed to evaluate the presence of anti-SARS-CoV-2 spike (S) receptor-binding domain (RBD) IgG over 8 months. A preliminary positive cutoff was set with the mean value of negative controls above 3 standard deviations.^1^ Neutralizing antibodies (NAbs) were measured by pseudovirus-based assays associated with two SARS-CoV-2 strains (S-D614 and S-G614) in 293T-ACE2 cells. The 50% inhibitory dose (ID_50_) was calculated as the NAb titer.

**Fig. 1:**
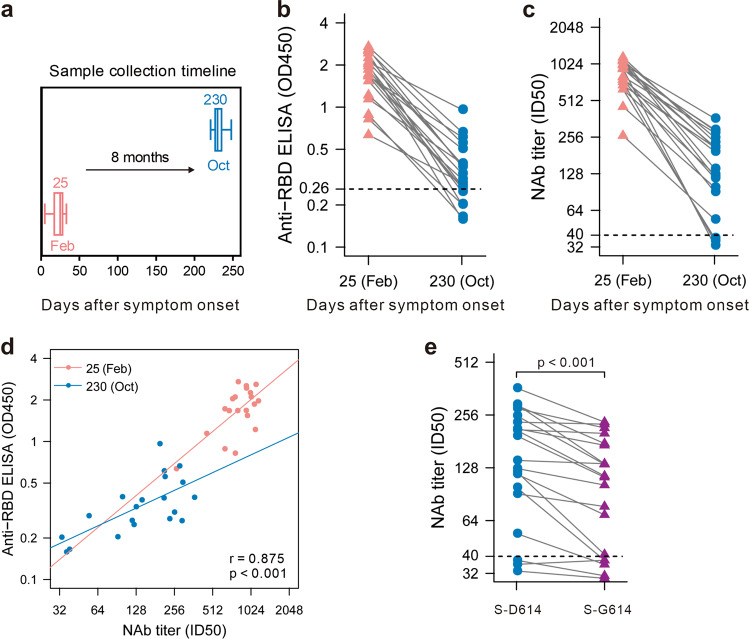
Maintenance of the humoral response to SARS-CoV-2 in convalescent patients over 8 months. **a** Blood samples were collected in February and October. Enzyme-linked immunosorbent assays (ELISAs) (**b**) and pseudovirus-based neutralizing assays (**c**) were performed to detect IgG levels and neutralizing antibody (NAb) titers against SARS-CoV-2. The thresholds of detection were 0.26 for the OD_450_ value and 1:40 for the ID_50_. **d** Correlation of IgG and NAb levels. **e** Neutralizing activities of convalescent plasma against SARS-CoV-2 S-D614 or S-G614 mutant at 8 months after symptom onset

Of the convalescent patients, there were 9 women and 11 men. The median age was 51.5 years (range 45–65). Except for two cases with severe symptoms, 90% of the infected patients had mild symptoms (Supplementary Table 1).

In all 20 participants, antibodies against the SARS-CoV-2 spike RBD decreased from a mean OD_450_ value of 1.78 (range 0.55–2.72) to 0.38 (range 0.15–1.01) over 8 months. When the OD_450_ value was <0.26, the specimen was considered seronegative. At follow-up time point 2 (in October), the IgG level of five participants (25%) had became negative (Fig. 1b). A similar decline was observed in the pseudovirus neutralization assay. Indeed, NAb titers decreased from a mean ID_50_ value of 836.55 (range 263–1160) to 170.30 (range 33–365). Among them, the NAb titers of three participants (15%) were lower than the threshold at 8 months after symptom onset (Fig. 1c). Moreover, NAb titers correlated significantly with IgG levels (*p* < 0.001) (Fig. 1d). The cross-protective role of NAbs at 8 months after symptom onset was evaluated by a pseudovirus-based neutralization assay using SARS-CoV-2 S-G614, which is currently the dominant strain worldwide. The NAb titers against the S-G614 mutant pseudovirus of five participants (25%) decreased below the threshold. Moreover, there was a statistically significant difference in the neutralizing efficacy of convalescent plasma against SARS-CoV-2 S-D614 and S-G614 mutant pseudoviruses (Fig. 1e).

Herein, we report changes in the humoral immunity response in SARS-CoV-2 convalescent patients over 8 months. In agreement with previous follow-up studies within a shorter time frame, declines in both IgG and NAb were observed.^1–3^ Furthermore, the better significant correlation between IgG and NAb levels in February than in October indicates that the anamnestic immune response and other protective immunity should be evaluated within the context of low levels of NAbs.^4^

Facing the challenge of the second wave of SARS-CoV-2 infection, the risk of reinfection among convalescent patients by the currently dominant strain (SARS-CoV-2 S-G614) is worth considering. Weaker neutralizing activity against the S-G614 mutant pseudovirus has been demonstrated. In two samples, NAb titers even quickly decreased from 1:99 or 1:122 to near the limit of detection. This might be a warning about the possible loss of protective capacity for convalescent plasma with lower titers against the SARS-CoV-2 S-G614 variant, similar to the reinfection case reported in Hong Kong.^5^ Therefore, more data about the longevity of humoral immunity are needed to evaluate the effectiveness of herd immunity.

## Supplementary information

Supplementary table 1
