# Supplementary material for: Changes in the humoral immunity response in SARS-CoV-2 convalescent patients over 8 months
Source: Cell Mol Immunol. 2021 Jan 8;18(2):490–1. doi: 10.1038/s41423-020-00605-4 (PMC7791534; doi:10.1038/s41423-020-00605-4)
Supplement: Supplementary file 1 — Supplementary table 1 [file 41423_2020_605_MOESM1_ESM.docx]

**Supplementary Table**

**Table 1.** Clinical characteristics of 20 convalescent individuals in the cohort^*^.

| Characteristics | Patients (N=20) |
| --- | --- |
| Age (median, IQR) | 51.5 (44.8-65.0) |
| Sex (Male/Female) |  |
| Male | 11 (55.0%) |
| Female | 9 (45.0%) |
| Exposure |  |
| From Wuhan | 3 (15.0%) |
| Close contacts | 17 (85.0%) |
| Severity |  |
| Non-severe | 18 (90.0%) |
| Severe | 2 (10.0%) |
| Hospitalization (days) | 25 (18-28) |
| Comorbidities |  |
| Hypertension | 4 (20.0%) |
| Cardiovascular disease | 2 (10.0%) |
| Diabetes | 3 (15.0%) |
| COPD | 1 (5.0%) |
| Chronic liver disease | 1 (5.0%) |
| Signs and symptoms |  |
| Fever | 7 (35.0%) |
| Fatigue | 1 (5.0%) |
| Dry cough | 6 (30.0%) |
| Inappetence | 1 (5.0%) |
| Myalgia | 1 (5.0%) |
| Dypnea | 2 (10.0%) |
| Expectoration | 4 (20.0%) |
| Pharyngalgia | 1 (5.0%) |
| Dizziness | 1 (5.0%) |
| Vomiting | 1 (5.0%) |
| Chill | 2 (10.0%) |
| Rhinorrhea | 1 (5.0%) |
| Chest stuffiness | 1 (5.0%) |

^*^Blood samples were obtained at Yongchuan Hospital of Chongqing Medical University, Chongqing, China
